# Haloboration: scope, mechanism and utility

**DOI:** 10.1039/d0nj02908d

**Published:** 2020-07-08

**Authors:** Sven Kirschner, Kang Yuan, Michael J. Ingleson

**Affiliations:** EaStCHEM School of Chemistry, University of Edinburgh Edinburgh EH9 3FJ UK michael.ingleson@ed.ac.uk

## Abstract

Haloboration, the addition of B–X (X = Cl, Br, I) across an unsaturated moiety *e.g.*, C=Y or C≡Y (Y = C, N, *etc.*), is dramatically less utilised than the ubiquitous hydroboration reaction. However, haloboration of alkynes in particular is a useful tool to access ambiphilic 1,2-disubstituted alkenes. The stereochemical outcome of the reaction is easily controlled and the resulting products have proven to be valuable building blocks in organic synthesis and materials chemistry. This review aims at providing the reader with a brief summary of the historic development and of the current mechanistic understanding of this transformation. Recent developments are discussed and select examples demonstrating the use of haloboration products are given with a focus on the major areas, specifically, natural product synthesis and the development of boron-doped polycyclic aromatic hydrocarbons (B-PAHs).

## Abstract 2

Haloboration is a mostly overlooked technique, yet it is a powerful way of transforming alkynes stereoselectively into difunctionalised ambiphilic alkenes, which readily undergo a plethora of highly useful subsequent reactions.

## Graphical abstract

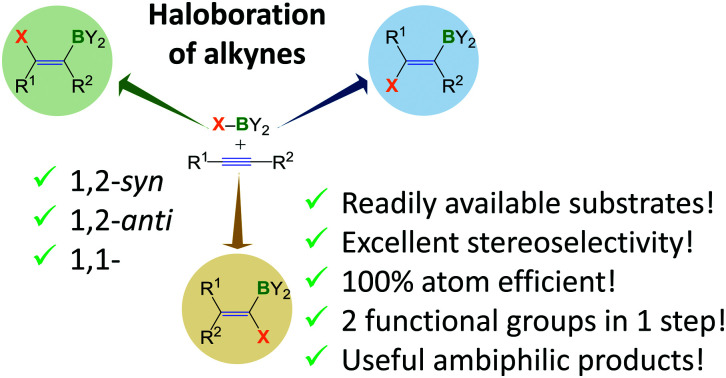

## 1. Introduction

Since the end of the last millennium, the principle of sustainability and atom economy increasingly has impacted the way scientific research is done.^1^ A chemical transformation that is 100% atom efficient is the addition reaction, in which a reagent is added across a multiple bond (*e.g.*, C≡C, C=C, or C=E, E: O, NR). One very important addition reaction is the Nobel Prize winning hydroboration reaction.^2^ Initially, it was almost exclusively used to access alcohols from alkenes by oxidative B–C bond cleavage. However, organic transformations like the Matteson homologation,^3^ Petasis variant of the Mannich reaction,^4^ Chan–Lam coupling,^5^ and, of course, the Suzuki–Miyaura reaction^6^ all utilise substrates that can be accessed by hydroboration reactions, rendering it a powerful tool in the synthetic chemist's toolbox. Unsurprisingly, researchers still strive to develop new hydroboration methods, *e.g.*, by expanding the scope or reducing the environmental impact.^7^ In contrast, haloboration – although discovered at roughly the same time – has remained a niche technique that has gained only a small fraction of the attention even though it adds an additional highly valuable group (a halide) in the same step. The concomitant installation of a boron unit and a halide generates functionality rich molecules, containing a nucleophilic C–B and an electrophilic C–X unit. Thus, it is surprising that this reaction is so under-utilised, despite being potentially useful to many.

In this review, we give a brief discourse of the historic development of the haloboration reaction, from the curiosity driven fundamental research mainly by the group of Lappert, to the usage of Pd catalysed cross-coupling reactions to demonstrate the full potential of haloboration from the group of Suzuki who broadened the scope and deepened the understanding of this reaction.^8^ We also provide an in-depth discussion of the underlying mechanism and select applications of the products from the haloboration reaction in the field of synthesis. Recently, haloboration has started to gain wider interest through its use in natural-product synthesis to introduce C=C double bonds stereoselectively, and in the synthesis of boron-doped polycyclic aromatic hydrocarbons (B-PAHs), thus these are the main applications focused on herein. B-PAHs are a relatively new class of organic materials with interesting optoelectronic properties and haloboration is a fast and convenient way to incorporate a borane unit and a halogen functionality into a PAH at the same time. Since the publication of the last reviews on haloboration, which were in the 1980s to the best of our knowledge,^8*d*^ the scope and utility of haloboration in synthetic chemistry has increased significantly. We hope that this review, focused on the use of boron electrophiles to haloborate C=Y and C≡Y nucleophiles (Y = C, N or O based substituents) facilitates the wider application of this useful, yet often overlooked, reaction.

## 2. Haloboration of simple alkynes using Y_2_B–X (Y = X or R)

### 2.1 The early work

**Scheme 1:**
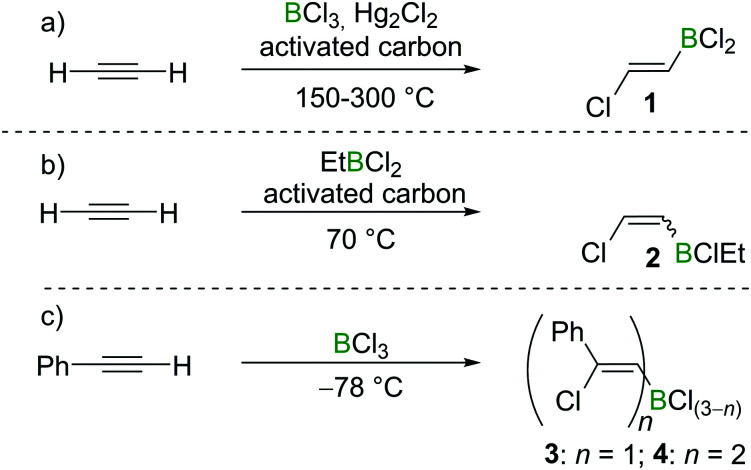
Chloroboration of acetylene/phenylacetylene under various conditions.

Chloroboration of alkynes was first explored by H. R. Arnold in 1946.^9^ With mercury(i) chloride on activated carbon as the catalyst, chloroboration of acetylene with boron trichloride (BCl_3_) was achieved to afford 2-chlorovinyldichloroborane at 150–300 °C (Scheme 1a). In this patent, the stereoselectivity of chloroboration was not determined. Subsequently, Jensen *et al.* repeated the synthesis under similar conditions and measured the dipole moment of the obtained product.^10^ The experimentally determined dipole moment (1.06(0.05) D) was found to be very close to the predicted value of *trans*-product **1** (1.05 D). For comparison, the dipole moment of the *cis*-isomer was predicted to be 3.23 D. Thus, *trans*-product **1** was believed to be formed. In addition, the *trans*-chloroboration product **1** was found to be significantly more stable than the *cis*-isomer by 145 kJ mol^−1^ in electronic energy based on *ab initio* calculations.

**Scheme 2:**
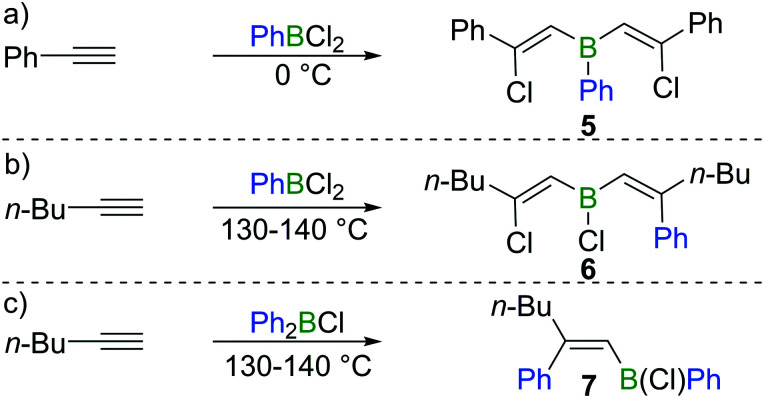
Chloroboration and carboboration of terminal alkynes.

Subsequent to Arnold's work, Gipstein *et al.* found that when EtBCl_2_ was used, in spite of the reduced Lewis acidity compared to BCl_3_, the chloroboration of acetylene in the presence of activated carbon could be realised at 70 °C, affording product **2** (configuration not determined) in 90% yield (Scheme 1b).^11^ Later, Lappert and co-workers studied the chloroboration reaction with a variety of alkynes and boranes.^12^ For instance, phenylacetylene was reported to undergo chloroboration with one equivalent of BCl_3_ readily even at −78 °C to afford the *syn*-addition product **3**. The obtained product **3** was shown to react with another equivalent of phenylacetylene in a *syn*-manner to give compound **4** (Scheme 1c). In the initial report by Lappert and co-workers, the configurations of **3** and **4** were assigned with incomplete evidence. Subsequent studies confirmed that chloroboration of terminal alkynes with BCl_3_ proceeds in a *syn*-manner.^13^ Although the reaction between BCl_3_ and terminal alkynes such as phenylacetylene occurs promptly, no reactivity was observed when internal alkynes such as diphenylacetylene and BCl_3_ were mixed at 15 °C. In accordance with the reactivity of BCl_3_, PhBCl_2_ readily reacted with two equivalents of phenylacetylene and compound **5** was obtained (Scheme 2a).^12^ In contrast, when 1-hexyne was treated with half an equivalent of PhBCl_2_, both chloroboration and carboboration occurred to give the product **6** (Scheme 2b). In a controlled reaction of 1-hexyne with Ph_2_BCl, carboboration occurred exclusively yielding compound **7** (Scheme 2c).

**Scheme 3:**
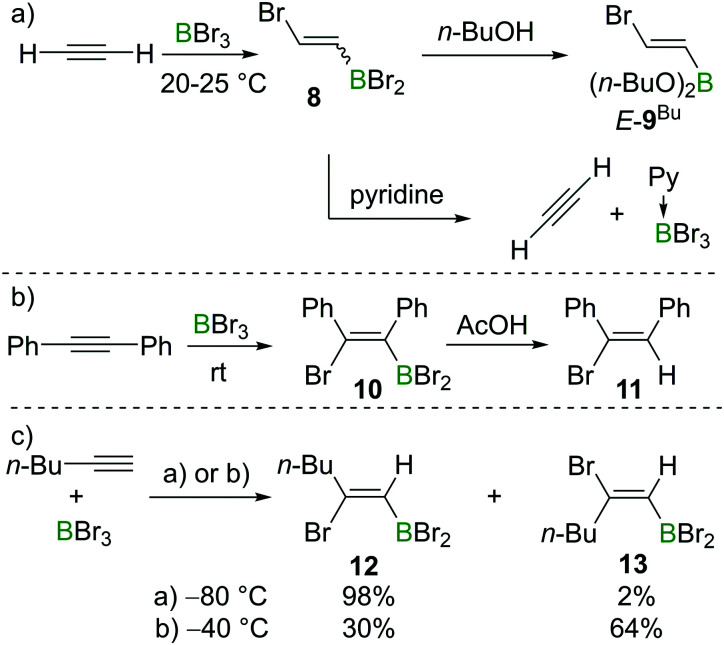
Bromoboration reactions to gain insight into the selectivity.

Lappert *et al.* also investigated the haloboration of acetylene using BBr_3_. The increased Lewis acidity of BBr_3_ (relative to BCl_3_) drastically facilitated the transformation which proceeded at room temperature and the addition product **8** was obtained (Scheme 3a). Alcoholysis of compound **8** with *n*-butanol afforded the known compound *E*-**9**^Bu^ with specified configuration. The configuration of *E*-**9**^Bu^ led the authors to assign a *trans*-configuration to **8**. However, a recent study suggested the bromoboration of acetylene typically gives a mixture of *E*/*Z* isomers at around 0 °C (for further details and mechanistic discussion, see Sections 2.2 and 2.3). Lappert *et al.* also found that the addition of pyridine (Py) to **8** resulted in the elimination of Py·BBr_3_ and regeneration of acetylene, which indicated that the bromoboration of acetylene might be reversible (Scheme 3a). The bromoboration of acetylene with BBr_3_ followed by esterification with alcohols provides a convenient route to halo-alkenylboronates that serve as versatile building blocks (*vide infra*).

Although BCl_3_ does not react with internal alkynes, Lappert and co-workers found that bromoboration of diphenylacetylene occurs readily in neat BBr_3_ at room temperature within one hour. In this case, *syn*-addition product **10** was formed as confirmed by subsequent protodeboronation reactions with acetic acid (AcOH) to give **11** (Scheme 3b), with protodeboronation known to proceed with retention.^12,14*a*^ Blackborow performed detailed studies on the bromoboration of 1-hexyne under various conditions (Scheme 3c).^14^ Generally, the bromoboration of the terminal alkyne proceeds in a Markovnikov fashion. The stereoselectivity, however, was found to be highly dependent on the reaction conditions. For example, when the reaction was performed at −80 °C in petroleum or dichloromethane, *syn*-addition product **12** was found to be dominant (98%) as determined by analysis post protodeboronation with AcOD. In contrast, when the reaction was performed at −40 °C, the stereoselectivity decreased with the major product being the *anti*-bromoboration product **13** (64%) while only 30% *syn*-addition product **12** was observed under these conditions. In addition to *E*/*Z* isomers, multiple haloborations to form the respective divinylbromoborane and trivinylborane also were observed. However, as the formation of these multiple borylation products is relatively slow under the reaction conditions and the product distribution was not well defined, these details are not discussed further here.

**Scheme 4:**
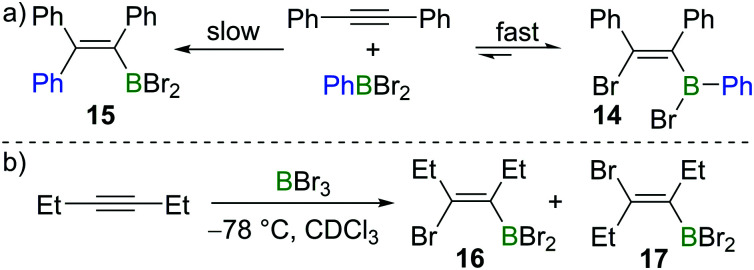
Isomerisation in reactions of internal alkynes with haloboranes.

Subsequent to Lappert's work with BBr_3_, Eisch and co-workers found that the less Lewis acidic borane PhBBr_2_ reacts with diphenylacetylene in a reversible manner (Scheme 4a).^15^ Upon mixing PhBBr_2_ and diphenylacetylene, *cis*-bromoboration product **14** was formed rapidly, which was confirmed by protodeboronation with acetic acid. However, prolonged storage of compound **14** in hydrocarbon solvents led to the irreversible formation of carboboration product **15**. This suggests that the bromoboration of diphenylacetylene with PhBBr_2_ is a kinetically favoured but reversible process while the carboboration is a slower and irreversible competing process. Wrackmeyer studied the reaction of 3-hexyne with BBr_3_ at −78 °C (Scheme 4b).^16^ When the sample was kept at room temperature for one hour, the *syn*-addition product **16** was found to be the major product in the reaction mixture (**16** : **17** = 15 : 1). However, after several days, the *E*-isomer **17** became dominant with the ratio of **16** : **17** switching to 1 : 7.

**Scheme 5:**
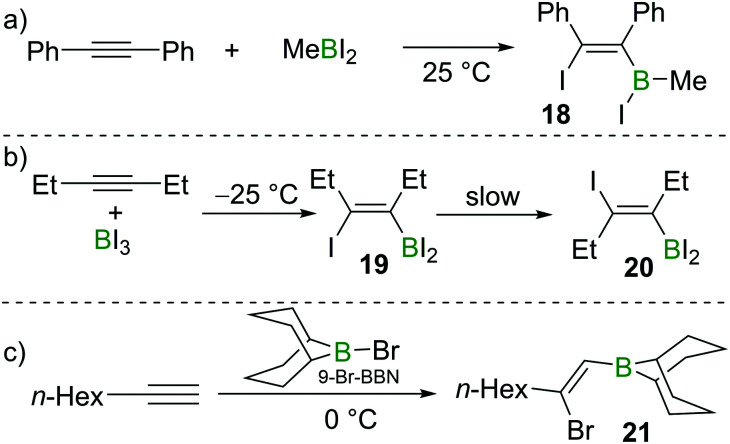
Haloboration reactions on various alkynes with different haloboranes.

Eisch also studied the reactivity of MeBI_2_ with diphenylacetylene. In this case, iodoboration occurred rapidly and compound **18** was obtained (Scheme 5a).^17^ Siebert found that iodoboration of 3-hexyne with BI_3_ furnished the *syn*-addition product **19** rapidly, which, in line with Wrackmeyer's observations, underwent slow isomerisation to form the *anti*-addition product **20** at room temperature (Scheme 5b).^18,19^

As discussed above, bromoboration of terminal alkynes with BBr_3_ does not occur exclusively in a 1 : 1 stoichiometry due to further reactions of the vinylBBr_2_ species with additional alkyne. One solution to this problem is to use 9-halo-9-borabicyclo[3.3.1]nonane (9-X–BBN; X = Br, I). Suzuki and co-workers found that 9-Br–BBN could react with one equivalent of 1-octyne to afford **21** in high regio- and stereoselectivity (Scheme 5c).^20^ They also found that 9-Br–BBN is inert to internal alkynes likely due to its lower Lewis acidity. These early studies clearly demonstrated the viability of alkyne haloboration, albeit complicated in many cases by formation of different haloboration isomers. The origin of *Z*- and *E*-configured haloboration products was at the time unclear and required subsequent DFT calculations to provide mechanistic insight.

### 2.2 Mechanistic studies

**Scheme 6:**
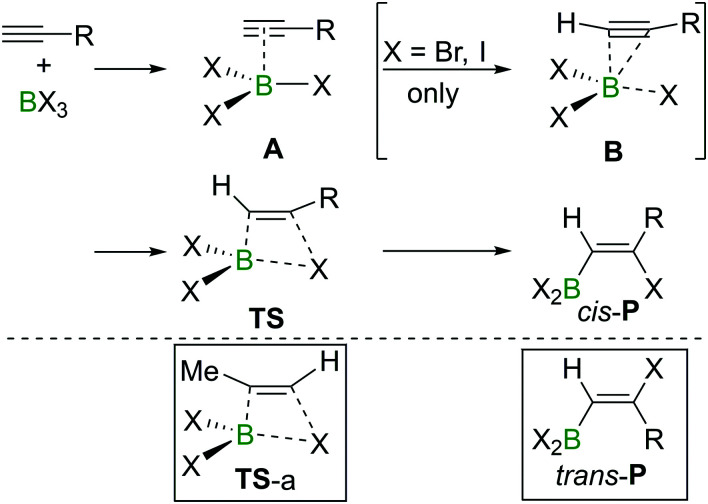
Reaction pathway for *cis*-haloboration of alkynes.

The haloboration of alkynes was investigated computationally initially by Uchiyama and co-workers.^21^*Ab initio* calculations with second-order Møller–Plesset perturbation theory (MP2) were performed on the haloboration of acetylene and propyne using a dichloromethane continuum solvent model (Scheme 6). The two alkynes first form loose van der Waals complexes **A** with BX_3_ (X = Cl, Br, I), which then may transform into π-bonded complexes **B** if X = Br or I but not Cl. Intermediates **A** (X = Cl) or **B** (X = Br, I) then convert into the *syn*-addition products *cis*-**P***via* four-centred transition state **TS**. Consistent with the observed reactivity of BX_3_ with alkynes (BBr_3_ > BCl_3_), the reaction energy barriers (energy of TS) decrease in order of BCl_3_ > BBr_3_ > BI_3_ for the *syn*-addition pathway.

**Scheme 7:**
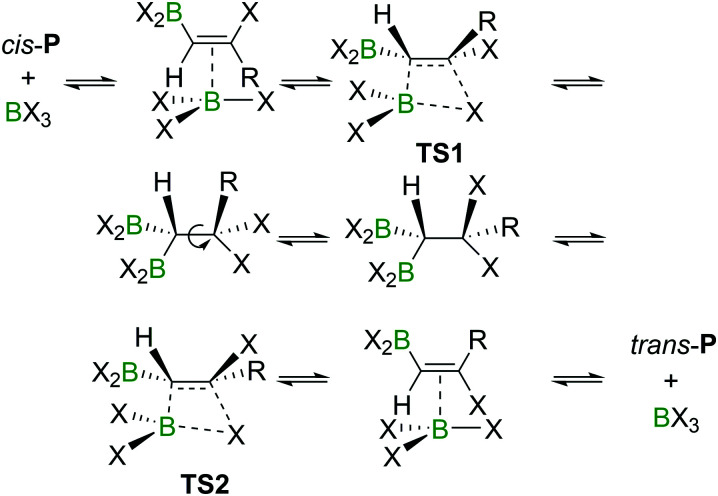
Possible mechanism for stereoconversion mediated by BX_3_.

An energy barrier of 33.8 kcal mol^−1^ was determined computationally for the *cis*-chloroboration of acetylene, which agrees with the experimental observations that chloroboration of acetylene required a high reaction temperature and a catalyst. In addition, the haloboration of propyne was found to have a lower energy barrier than acetylene, which is ascribed to hyperconjugation stabilising the developing positive charge at carbon in **TS** (when R = Me). Furthermore, the *anti*-Markovnikov pathway for the chloroboration of propyne was also explored computationally. The transition state of *anti*-Markovnikov pathway **TS**-a (X = Cl, Δ*G*^‡^ = 35.6 kcal mol^−1^) was found to be much higher than the Markovnikov pathway (X = Cl, Δ*G*^‡^ = 15.5 kcal mol^−1^). The results fit well with the high regioselectivity of terminal alkyne haloboration reactions. For all transformations, the *trans*-haloboration products *trans*-**P** also were computed. For acetylene, the*y* were found to be thermodynamically more stable than the *cis*-products, which is in agreement with the observation that the *trans*-chloroboration product was formed exclusively at high temperature in the aforementioned reports (*cf.* Section 2.1). In contrast, the *syn*-addition products of propyne haloboration are very close in energy to their *anti*-addition isomers. Again, this result is consistent with the observation that bromoboration of 1-hexyne at temperatures above −40 °C gave a mixture of both isomers. After the exploration of several different potential reaction pathways, Uchiyama and co-workers proposed that the stereoconversion proceeded *via* a haloboration/retro-haloboration mechanism of *cis*-**P** with BX_3_ (Scheme 7).

The chloroboration of the internal alkyne 2-butyne, and propene was also investigated. For both substrates, the chloroboration was found to be endergonic at 293 K (Δ*G* = 2.8 kcal mol^−1^ for 2-butyne; Δ*G* = 9.4 kcal mol^−1^ for propene) consistent with that lack of reactivity between BCl_3_ and internal alkynes and olefins.

### 2.3 Recent studies into the bromoboration of simple alkynes with BBr_3_

**Scheme 8:**
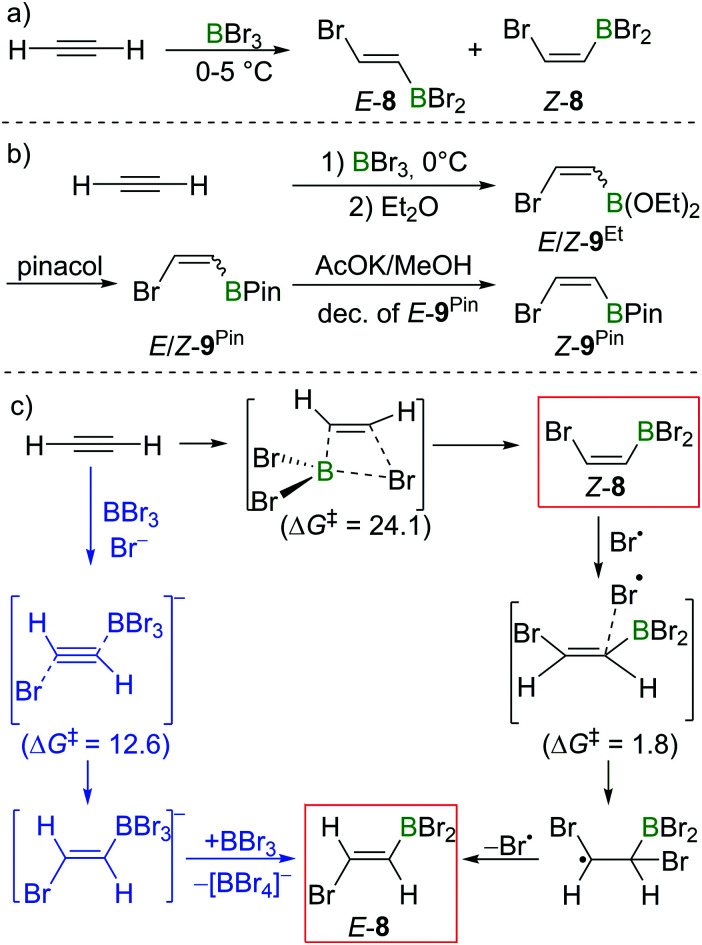
(a) Reaction of acetylene with BBr_3_. (b) Brønsted-acid-free workup conditions allow for the selective isolation of *Z*-**9**^Pin^. (c) Possible reaction pathways and energies (kcal mol^−1^) of acetylene bromoboration.

In Lappert's initial studies, the stereoselectivity for the bromoboration of acetylene was determined by analysis of the post-esterification (conversion of C–BBr_2_ to C–B(OR)_2_) products. Recently, Mazal and co-workers carried out similar haloboration experiments at 0 °C and monitored the formation of *E*/*Z* vinylbromoborane **8** by NMR spectroscopy (Scheme 8a). With BBr_3_ distilled from Mg turnings, a mixture of *E*/*Z* isomers was obtained (*E* : *Z* = 15 : 85). Interestingly, addition of small amounts of water, NEt_3_ or [*n*-Bu_4_N]Br to the bromoboration reaction facilitated the formation of *anti*-addition product *E*-**8**. It is even more notable that the authors exclusively found the *trans*-vinylboronate *E*-**9**^Pin^ post esterification workup. They proposed radical or polar addition mechanisms involving adventitious HBr to rationalise the observed reactivity.^22^ By modifying the workup procedures to avoid the formation of HBr, a mixture of *E*/*Z*-bromovinylboronate *E*/*Z*-**9**^Et^ could be obtained, which was subjected to transesterification with pinacol, yielding *E*/*Z*-**9**^Pin^. Selective decomposition of the *E*-isomer (AcOK, MeOH) enabled the isolation of *Z*-bromovinylboronate *Z*-**9**^Pin^ in useful yields (Scheme 8b).

In their proposed radical mechanism, a *cis*-bromoboration of acetylene occurs first to give *Z*-**8** with an energy barrier of 24.1 kcal mol^−1^ (MP2/6-31+(d)/PCM(DCM)). Attack of a bromine radical (proposed to be generated from adventitious HBr) on *Z*-**8** proceeds with almost no barrier (1.8 kcal mol^−1^). Bond rotation followed by bromine radical elimination was then assessed to afford the *trans*-bromoboration product *E*-**8**. An *anti*-addition of BBr_3_ and Br^−^ to acetylene was also suggested for the formation of *E*-**8**. Acetylene was calculated to first form a π-bonded complex with BBr_3_, which was then attacked by Br^−^ to form a borate intermediate. Abstraction of Br^−^ with BBr_3_ from the intermediate gave *E*-**8**. The energy barrier for this process was determined to be 12.6 kcal mol^−1^.

### 2.4 1,1-Bromoboration of internal alkynes

**Scheme 9:**
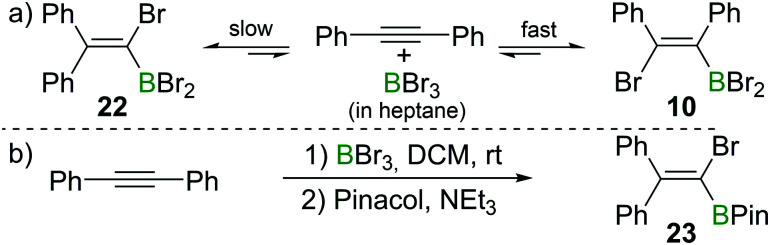
1,1-Bromoboration of diphenylacetylene yields mixtures of the *syn*-1,2-addition product **10** and the 1,1-addition product **22**.

Recently, the group of Ingleson have revisited the reaction of internal alkynes with BBr_3_. Consistent with Lappert's observations, they found that in non-polar solvents such as heptane, the reaction of diphenylacetylene with BBr_3_ at room temperature afforded the *cis*-1,2-addition product **10** within a few minutes.^23^ However, upon heating to 60 °C or prolonged standing at room temperature, the 1,1-addition product **22** was observed (Scheme 9a). When a polar solvent such as dichloromethane was used in the haloboration reactions, both **10** and **22** were formed within a few minutes with **22** being the major product (Scheme 9b). No *trans*-1,2-bromoboration product was observed by *in situ* NMR spectroscopy at any stage of the reaction. These observations suggest that 1,1-bromoboration proceeds through a polar transition state, likely a vinyl cation type intermediate(s) which is more stabilised by the more polar solvent dichloromethane. Upon reaction with pinacol and NEt_3_, compound **22** was converted into the corresponding pinacol ester **23** while **10** was converted back into diphenylacetylene. The 1,1-bromoboration could be extended to diarylalkynes and arylalkylalkynes, providing a convenient way to 1-bromo-2,2-diaryl substituted vinylboronate esters.

This is a further example that the reactions of alkynes and BX_3_ can give conditions dependent outcomes. Nevertheless, the use of the appropriate conditions and work-up can lead to isolation of a single haloboration product in useful yield. This diversity in outcome (1,1-, *syn*-1,2- or *anti*-1,2-haloboration products being accessible) arguably increases the power of the haloboration transformation.

## 3. Other alkyne haloboration reactions

This section covers recent synthetic reports that use other (*i.e.*, not Y_2_B–X, Y = X or R) boron electrophiles or provides alkyne haloboration products that are distinct to those reported in the work discussed in Section 2.

### 3.1 Borocation mediated chloroboration of alkynes

**Scheme 10:**
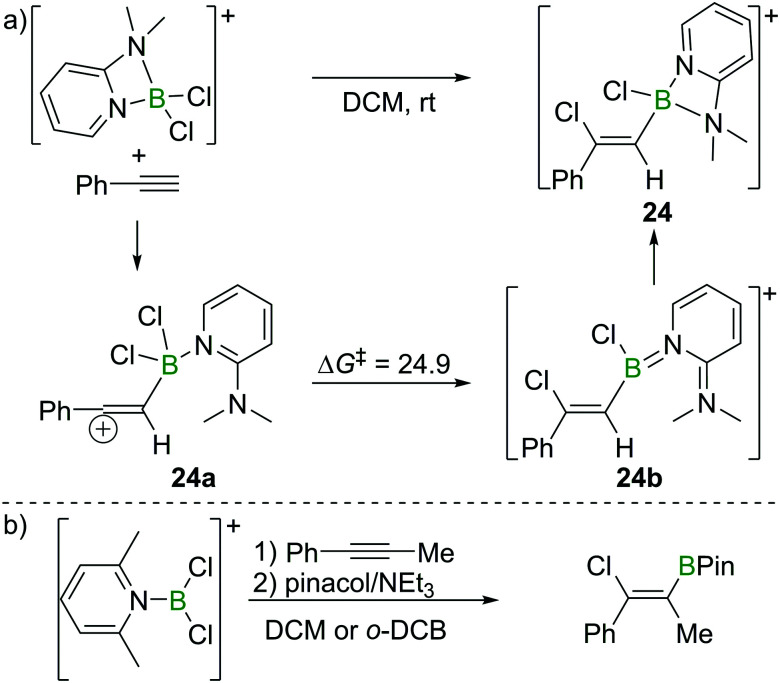
Reaction route and calculated energies (kcal mol^−1^) for chloroboration of phenylacetylene with boronium/borenium complexes.

As discussed in Section 2.2, Uchiyama and co-workers found the chloroboration of internal alkynes with BCl_3_ to be thermodynamically uphill. However, more electrophilic boranes such as BBr_3_ and BI_3_ react with internal alkynes readily. Therefore, Ingleson *et al.* envisioned the use of more electrophilic chloroborane species, such as borocations, to enable internal alkyne chloroboration.^24^ They prepared the boronium (tetracoordinate at B mono-cation) salt [Cl_2_B(2-DMAP)][AlCl_4_] (2-DMAP = 2-dimethylaminopyridine) by sequential addition of 2-DMAP and AlCl_3_ to BCl_3_ (Scheme 10a). Due to the strain within the four-membered boracycle, this complex showed a low energy barrier to ring opening and reacted as a masked borenium (= tricoordinate B monocation) ion. [Cl_2_B(2-DMAP)][AlCl_4_] reacted with one equivalent of a terminal alkyne at room temperature with high regio- and stereoselectivity. In contrast to the haloboration of alkynes with neutral BX_3_ which proceeded *via* a four-membered transition state with concerted formation of B–C and Cl–B bonds, chloroboration of phenylacetylene with [Cl_2_B(2-DMAP)][AlCl_4_] was calculated to proceed *via* a vinyl-cation intermediate, **24a**. Intramolecular chloride transfer in **24a** occurs with an energy barrier of 24.9 kcal mol^−1^ to give **24b** (at the M06-2X/6-311G(d,p)/PCM(DCM) level of theory), which then rearranges to afford the chelated compound **24**. Although the reaction between the boronium complex and terminal alkynes was facile, no reactivity was observed between [Cl_2_B(2-DMAP)][AlCl_4_] and internal alkynes. This is presumably due to the requirement to open the four-membered boracycle within [Cl_2_B(2-DMAP)]^+^ prior to the haloboration reaction, coupled with the significant N → B π donation (as shown in **24b**) in the borenium isomer of [Cl_2_B(2-DMAP)]^+^ (reducing the Lewis acidity at B) leading to unfavourable energetics.

Ingleson and co-workers also reported that when the borenium salt [Cl_2_B(Lut)][AlCl_4_] (Lut = 2,6-lutidine) was used, *syn*-addition of the B–Cl bond across both terminal and internal alkynes occurred (Scheme 10b), presumably due to the enhanced electrophilicity at B in this borocation relative to the 2-DMAP analogue. Good chloroboration stereoselectivity was achieved for dialkyl, diaryl and arylalkylalkynes. Remarkably, for arylalkylalkynes, regioselective chloroboration could also be readily realised. All the products could be converted into the corresponding pinacol boronate esters by subsequent esterification with no loss in stereo-/regioisomeric purity.

### 3.2 1,2-*trans*-Chloroboration of alkynes

**Scheme 11:**
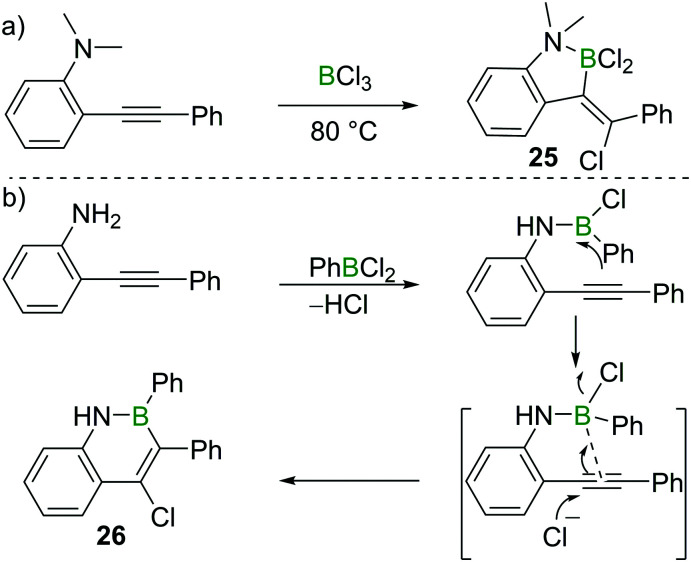
Amino group directed *trans*-chloroboration of internal alkynes.

Chloroboration of alkynes typically proceeds in a *syn*-manner. The group of Ingleson found that treatment of 2-dimethylaminotolan with BCl_3_ gave the unusual *anti*-addition product **25** (Scheme 11a).^25^ Subsequently, Pei and co-workers reported that 2-aminotolan also reacted with PhBCl_2_ to give the *trans*-chloroboration product **26** (Scheme 11b).^26^ The reaction was proposed to be initiated through the activation of the triple bond by the boron moiety, which was then followed by a nucleophilic attack of the alkyne with chloride. This method serves as a convenient way to prepare B,N-fused polycyclic aromatic hydrocarbons. The disparity between the formation of five membered **25** and six membered **26** is notable. This can be attributed to the all sp^2^-containing **26** having a strong preference for forming six membered boracycles *via* electrophilic borylation, while the incorporation of a single tetrahedral centre (as in **25**) leads to five membered boracycles from electrophilic borylation being the favoured products.^27^

### 3.3 1,3-Chloroboration of alkynes

**Scheme 12:**
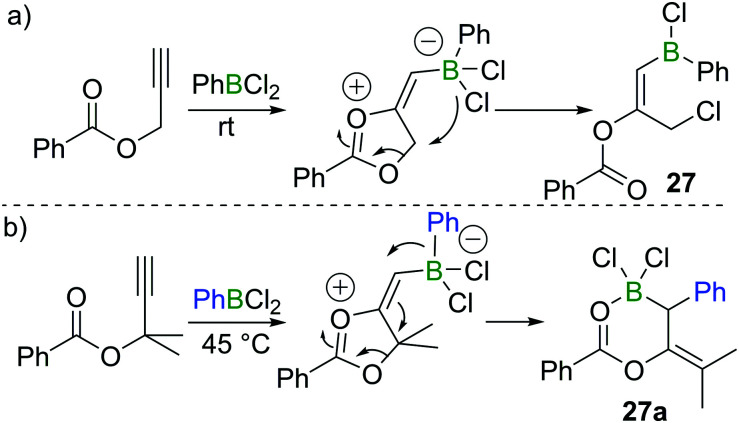
1,3-Chloroboration/1,1-carboboration of propargyl esters.

In 2018, Melen *et al.* reported a unique 1,3-chloroboration of propargyl esters.^28^ By treating propargyl benzoate with 1 equivalent of PhBCl_2_, an intermediate dioxolanonium ion was proposed to be formed *via* boron promoted cyclisation. The intermediate was assumed to subsequently undergo ring-opening and chloride migration to furnish the corresponding product **27** in high yield at room temperature (Scheme 12a). Interestingly, for a related propargyl ester with two methyl groups at the propargylic position, 1,1-carboboration occurred when PhBCl_2_ was added to give **27a** (Scheme 12b), presumably due to dimethyl substituents leading to a higher barrier for the 1,4-chloride transfer.

### 3.4 Chloroboration of diynes

**Scheme 13:**
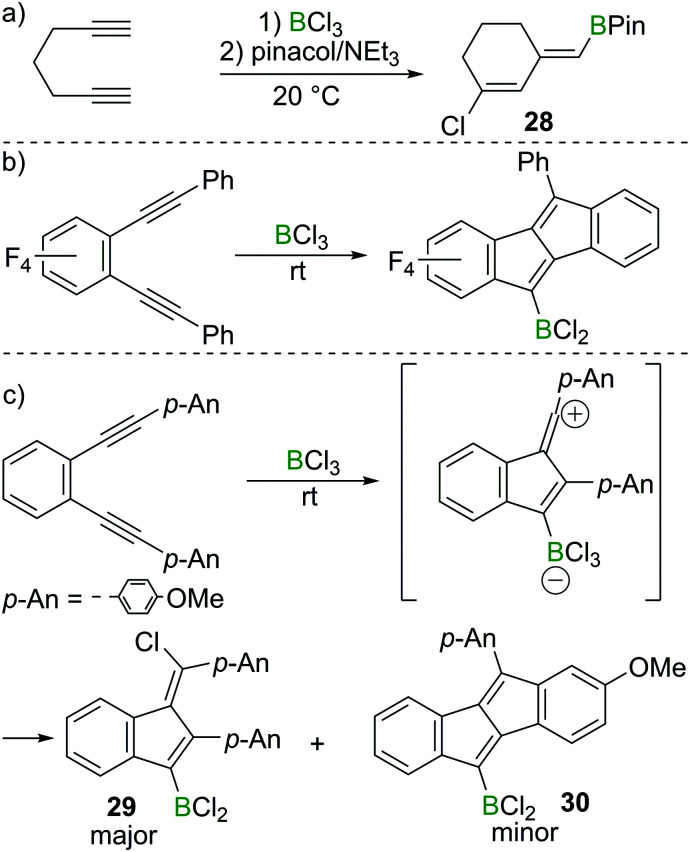
Borylative cyclisation/chloroboration of diynes.

Ingleson and co-workers found that 1,6-heptadiyne reacts rapidly with BCl_3_ at room temperature to afford a chlorinated cyclohexene featuring an exocyclic vinylBCl_2_ moiety.^29^ The corresponding pinacol protected compound **28** could be isolated in high yield post esterification with pinacol/NEt_3_ (Scheme 13a). In this case terminal alkyne 1,2-haloboration must have a higher barrier than intramolecular reaction of the alkyne–BCl_3_ adduct with the second alkyne. Furthermore, the reaction outcome was highly solvent dependent, with dichloromethane and dichloroethane affording **28** in good yield, whereas the use of chloroarenes resulted in very low yields of **28**, suggesting the vinyl chloride is made *via* a carbocationic intermediate, which can engage in side-reactions with aromatic solvents.

Although BCl_3_ shows no reactivity towards internal alkynes, facile transformations between BCl_3_ and 1,2-dialkynyl benzenes have been reported by the groups of Erker, Yamaguchi and subsequently Ingleson.^30,31^ Presumably these proceed from the adduct between the internal alkyne and BCl_3_. In these reports, dibenzopentalenes or analogues were the major products in most cases (Scheme 13b). However, Ingleson *et al.* found that by introduction of electron donating groups such as *p*-methoxyphenyl (*p*-An) to 1,2-dialkynyl benzene substrates, the major product is the benzofulvene **29***via* a formal 1,4-chloroboration reaction. In this case the dibenzopentalene **30** is only observed as the minor product (Scheme 13c).^31^

**Scheme 14:**
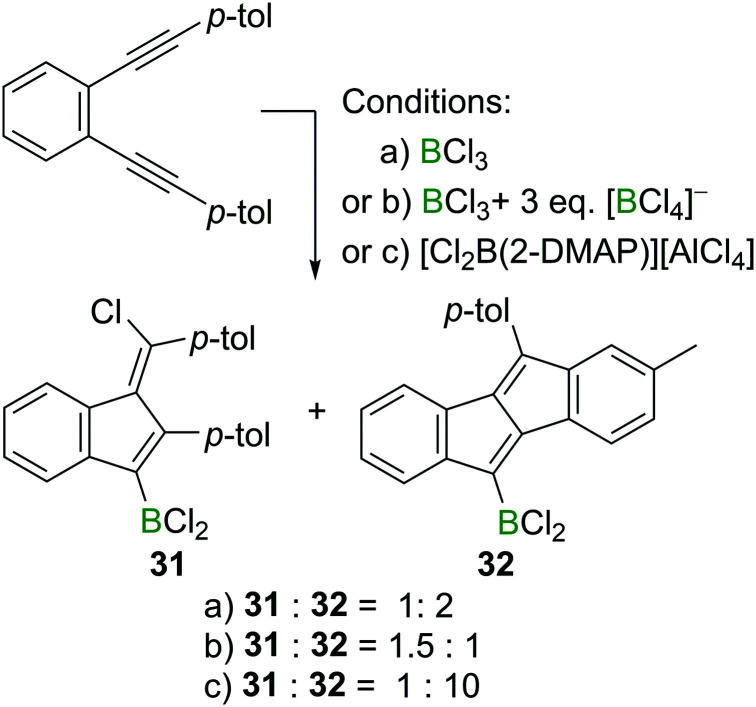
Change in product ratio on altering the boron electrophile or by the addition of an exogenous chloride source.

Interestingly, the formation of 1,4-chloroboration products becomes favoured in other cases by the addition of an exogenous chloride donor such as [BCl_4_]^−^ (Scheme 14). For example, 1,2-bis(*p*-tolylethynyl)benzene reacts with BCl_3_ affording the corresponding benzofulvene **31** and dibenzopentalene **32** in a 1 : 2 ratio. However, in the presence of three extra equivalents of a [BCl_4_]^−^ salt, the ratio of **31** and **32** switched to 1.5 : 1, indicating the role of [BCl_4_]^−^ in promoting the formal chloroboration reaction potentially by transferring chloride to the vinyl cation zwitterionic intermediate. Furthermore, precluding the presence of [BCl_4_]^−^ or [RBCl_3_]^−^ species in the reaction mixture by using boronium salt [Cl_2_B(2-DMAP)][AlCl_4_] significantly reduced the amount of the 1,4-chloroboration product observed. These studies therefore reveal that in the haloboration of diynes the halide source must be considered carefully to ensure a successful reaction outcome.

### 3.5 Directed *trans*-bromoboration of alkynes

**Scheme 15:**
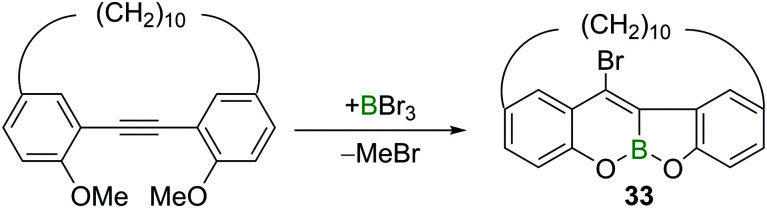
Directed *trans*-bromoboration of internal alkynes.

In addition to the aforementioned *trans*-bromoboration of acetylene and terminal alkynes, there are a limited number of other examples of internal alkyne *trans*-bromoboration reactions. Yamato and co-workers reported a BBr_3_ induced transformation of a *o*,*o*′-dimethoxy-substituted tolan derivative (Scheme 15).^32^ The highly Lewis acidic BBr_3_ induced a twofold ether cleavage and intramolecular *trans*-bromoboration, yielding benzofurochromene derivative **33**.

Pei and co-workers also reported a directed *trans*-bromoboration similar to their *trans*-chloroboration reaction discussed above (*cf*. Scheme 11).^26^ Due to the similarities the details are not discussed again herein.

Sections 2 and 3 show the utility of alkyne haloboration, and this is by far the most developed reaction. The application of the haloboration reaction to other π systems is much less developed and the limited examples reported currently to the best of our knowledge are discussed in Section 4.

## 4. Haloboration of C=C double bonds

**Scheme 16:**
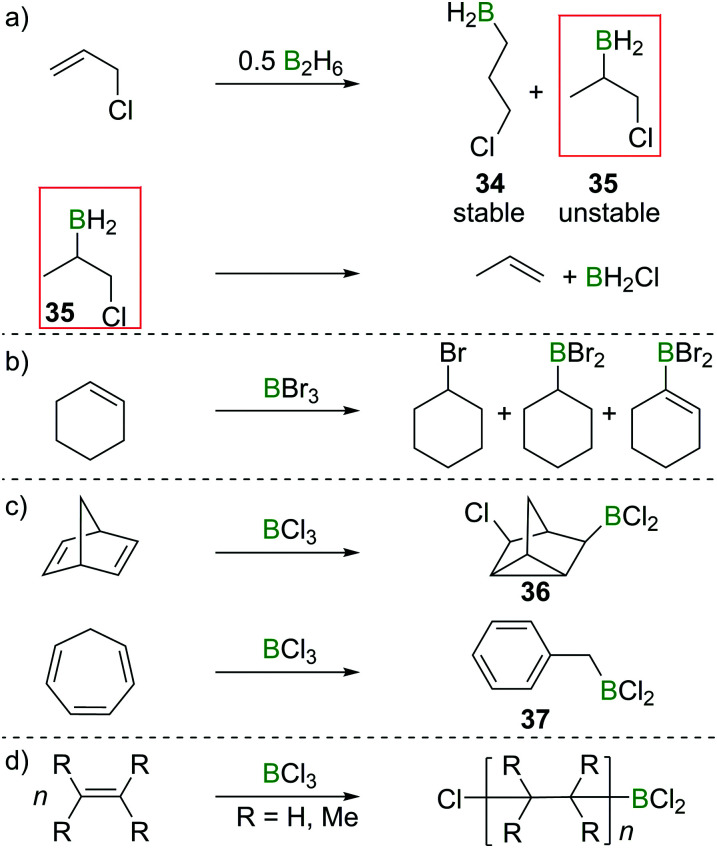
(a) Hydroboration of allyl chloride gives 3-chloropropylborane (**34**) and 1-chloro-2-propylborane (**35**); the latter decomposes under elimination of BH_2_Cl to give propene. (b) Reaction of cyclohexene with BBr_3_ yields a mixture of products. (c) and (d) Select examples of BCl_3_ initiated cationic rearrangements or polymerisation of alkenes.

In their attempted hydroboration of vinyl chloride with B_2_H_6_ at −80 °C, DuPont *et al.* observed decomposition of the putative tris(2-chlorovinyl)borane upon warming to room temperature, whereas allyl chloride underwent hydroboration smoothly even at ambient conditions.^33^ Those findings were confirmed by Brown and Köster, who investigated the selectivity of allyl chloride hydroboration. Hydroboration resulted in a formal 6 : 4 *anti*-Markovnikov (**34**)/Markovnikov (**35**) selectivity. However, the Markovnikov product underwent rapid elimination of the vicinal BH_2_ and Cl groups to form H_2_BCl and propene (Scheme 16a).^34,35^ These observations foreshadowed the subsequent reports that found formation of olefin 1,2-haloboration products to be an energetically unfavourable process. Haloboration experiments on cyclohexene by Lappert *et al.* supported olefin haloboration being energetically uphill: only a mixture of products could be identified, with no α-haloalkyl borane (the primary product from haloboration) observed, indicating that additional reactivity has to take place to lead to an overall exergonic process (Scheme 16b).^36^

Rearrangements also can be used to trap initial olefin haloboration products, for example the reaction of BCl_3_ with suitable olefins such as norbornadiene or cycloheptatriene, furnishes tricyclene **36** or BnBCl_2_ (**37**, Scheme 16c).^36,37^

**Scheme 17:**
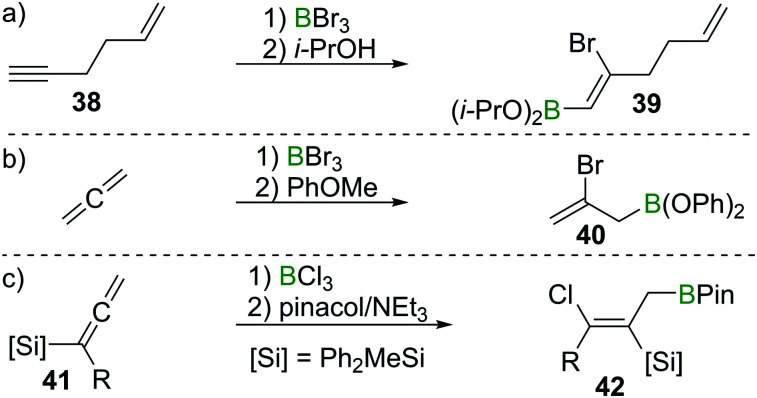
Select examples of selectivity of alkyne haloboration over alkene haloboration and of haloboration reactions on C=C double bonds.

These observation are consistent with the ability of boron trihalides to function as initiators in alkene polymerisation (Scheme 16d), instead of resulting in simple olefin haloboration.^38^ These experimental findings were corroborated through the earlier discussed calculations by Uchiyama *et al.*, who showed that haloboration of alkenes is thermodynamically unfavourable.^21^ This is further supported by the experimental finding that alkynyl substituted alkene **38** reacts selectively with BBr_3_*via* the alkyne moiety to yield **39** (Scheme 17a).^39^ Thus haloboration of olefins is an uncommon route to β-haloalkylboranes with only one inter- and one intramolecular synthesis reported to our knowledge.^40,41^ Other methods are preferred to prepare this versatile structural motif.^42^

While isolated olefins do not undergo haloboration, Lappert *et al.* showed that allenes reacted smoothly at −20 °C with BBr_3_ to give the respective 1,2 adduct. Isolation of the bromoborane, however, was problematic as polymerisation of the remaining alkene function occurred at elevated temperatures during attempted distillation.^36^ By esterification of the BBr_2_ group through reaction with anisole, polymerisation was prevented and the adduct could be isolated (as **40**), thus providing stereoselective access to allylboronic esters (Scheme 17b).^43^ Iodoboration of terminal allenes with 9-I–BBN also can be a useful route to 2-iodoalkenes, if post haloboration the 9-BBN moiety is removed *via* acetolysis.^44^ Notably, if acetolysis is omitted, this reaction can furnish a sought after allylboronate without the need for allyl-metal species which are prone to 1,3 metallotropic shifts.^45^ If silylated allenes such as **41** are employed, the outcome of the reaction changes. Instead of the 1,2 haloboration product, the formal 1,3 haloboration product **42** is obtained (Scheme 17c). Quantum chemical calculations suggest silyl migration followed by sterically driven 1,3 boryl shifts to be responsible for this special stereoselectivity.^46^

## 5. Haloboration of E=C bonds (E=O, NR)

**Scheme 18:**
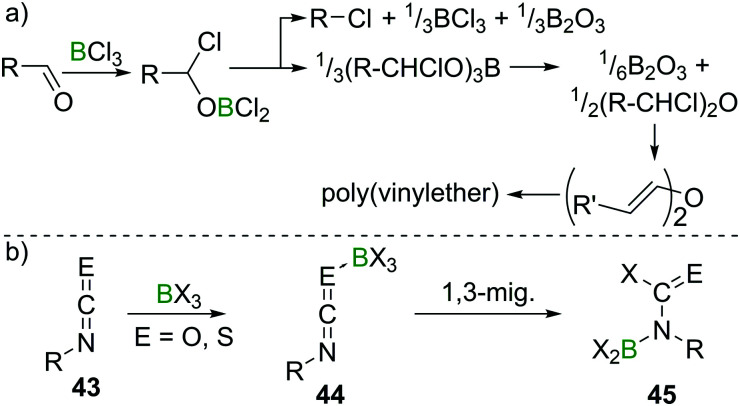
Reaction of aldehydes with BCl_3_ leading to complex mixtures, depending on the nature of the aldehyde. With iso(thio)cyanates, well defined products are obtained.

Lappert *et al.* investigated the reactivity of aldehydes towards BX_3_ and suggested haloboration across C=O to be a first step in a cascade of reactions that, depending on the nature of the aldehyde, eventually lead to borate esterification, formation of alkylhalides, haloalkylethers, or enolisation with concomitant polymerisation of the resulting vinylboric ester or alkenylether (Scheme 18a).^47^ The reaction of acetone with BCl_3_, however, only yielded an ill-defined mixture of products upon release of HCl, which precluded characterisation.^48^ Remarkably, when perhalogenated ketones (Hal = F, Cl, Br) were employed, usually haloboration of the C=O bond occurred,^49^ but in some instances, simple halide exchange is favoured, giving a different boron halide and the respective perhaloketone.^50^ In the case of isocyanates and isothiocyanates (**43**), multiple C=E double bonds exist. In these cases, haloboration proceeds stepwise *via* an R–N=C=E → BX_3_ (E = O, S) adduct **44**, followed by 1,3 migration of X to C. The higher stability of a C=O bond compared to a C=N bond then leads to a B shift, giving aminoborane derivatives **45** (Scheme 18b).

**Scheme 19:**
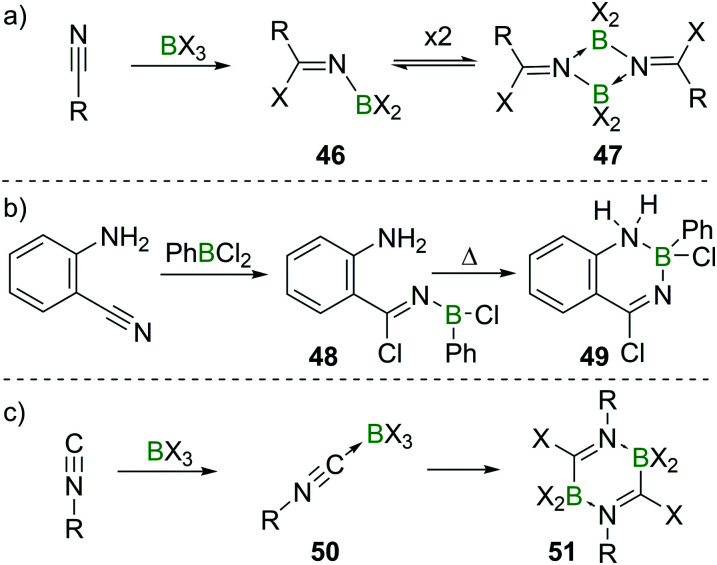
Select examples of E=C haloboration reactions.

Nitriles, which are isostructural and isoelectronic to alkynes can react with BX_3_ in a fashion related to their C≡C analogues. However, due to the lone pair at the N atom, the resulting aminoboranes **46** exist in an equilibrium with their cyclic dimers (**47**, Scheme 19a).^51,52^ In aminobenzonitriles, reactivity depends on the position of the two groups relative to each other. For *para*- and *meta*-substituted aminobenzonitrile, simple adducts of the type R–NH_2_ → BX_3_ form. In the case of *ortho*-aminobenzonitrile and PhBCl_2_, haloboration of the C≡N triple bond occurs, furnishing aminoborane **48** which at elevated temperatures cyclises to give the heterocyclic product **49** (Scheme 19b).^53^ In contrast to the *meta*- and *para*-substituted benzonitriles, ambidentate cyanamide reacts exclusively *via* haloboration of the C≡N triple bond giving dimeric iminoboranes, due to the strong and immediate mesomeric effect of the NH_2_ group on the C≡N triple bond and the thereby increased basicity of the cyano N-atom.^54^ Isonitriles form stable adducts **50** with the higher haloboranes BX_3_ (X = Cl, Br, I; for X = F, polymerisation occurs). If heated, these compounds undergo 1,1-haloboration and dimerisation to form 2,5-dihydrodiborapyrazines (**51**, Scheme 19c).^55^

**Scheme 20:**
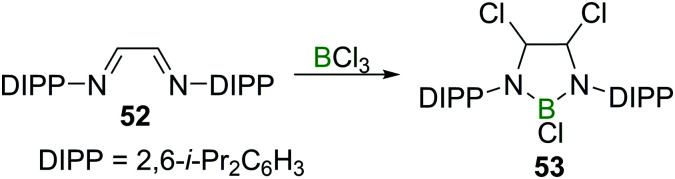
Chloroboration of 1,4-diazabutadienes to access 1,3,2-diazaborolidines.

Finally, reports on the reaction of BX_3_ with C=NR double bonds are, just like in the case of C=C double bonds, scarce, and limited to either highly electron deficient perfluorinated imines^56,57^ or chelating 1,4 diazabutadienes such as **52**.^58^ The latter is an early example of a simple route to 1,3,2-diazaborolidines (**53**, Scheme 20), which play a crucial role in accessing nucleophilic boranes.^59^

## 6. Application of alkyne haloboration products in organic synthesis

As described in Sections 2 and 3, haloboration of terminal alkynes is a versatile tool to access selectively 1,1- or 1,2-difunctionalised alkenes. The stereoselectivity can be controlled and the resulting vinylboranes show ambiphilic reactivity: the halogenated C atom is a potential electrophile, whereas the boron-bonded C atom behaves as a nucleophile. In this section we focus on applications that take advantage of both these groups or use the electrophilic vinylBX_2_ intermediate (particularly in reactions other than esterification). The applications highlighted are distinct to the plethora of reports on forming and utilising vinyl-boronate esters, and the reader is directed to the excellent recent reviews on these topics.^60,61^

**Scheme 21:**
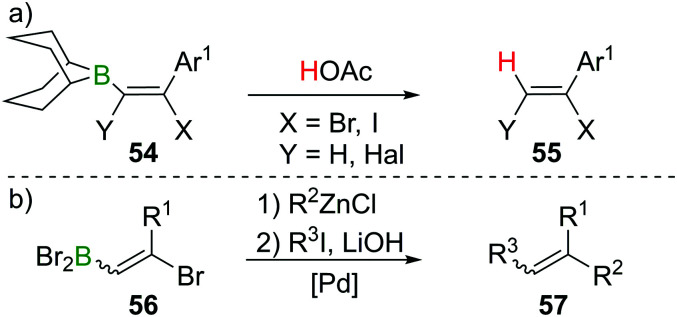
Early examples of functionalisation *via* B terminus.

Haloboration initially was used to stereoselectively produce singly or doubly halogenated terminal alkenes by reacting either terminal alkynes or haloalkynes with *e.g.*, 9-Br–BBN^13,14,62^ to give the intermediate alkenylborane **54** followed by acetolysis (**55**, Scheme 21a).^63–65^ 1,2-Dihaloalkenes can also be accessed from terminal alkynes if post haloboration the boron moiety is transformed into an R–BF_3_K salt and then treated with an electrophilic halogenating agent.^66^ Suzuki *et al.* first employed vinylBBr_2_ derivative **56** in a one-pot two-steps Negishi/Suzuki–Miyaura cross-coupling sequence to access 1,2-disubstituted alkenes (**57**) selectively (Scheme 21b).^67,68^ Although yields were good, the β-bromoalkenyl dibromoboranes **56** were found to be prone to retro-haloboration in the presence of Pd complexes, making further derivatisation to boronic esters expedient to lower the Lewis acidity of the B atom.^69–71^ During their fundamental studies, Lappert *et al.* used esterification of R–BX_2_ with catechol or alkanols to transform their products into stable and conveniently analysed derivatives.^12,37^ Esterification by ether cleavage^72^ or by reaction with the respective alcohol^73^ gave boronic esters with prolonged shelf-life.^25^ Other functionalisation includes formation of R–B(dan) (dan = 1,8-diaminonaphthalen-*N*,*N*′-diyl),^74^ R–B(MIDA) complexes (MIDA = *N*-Methyliminodiacetate),^75^ or R–BF_3_K salts.^66,76^ As expected, these classes of compounds are versatile reagents in transition-metal catalysed transformations such as Suzuki–Miyaura cross-coupling or Rh-catalysed [2 + 2 + 2] cycloadditions owing to their ambiphilic nature (electrophilic C–X/nucleophilic C–B).^39,77–80^

**Scheme 22:**
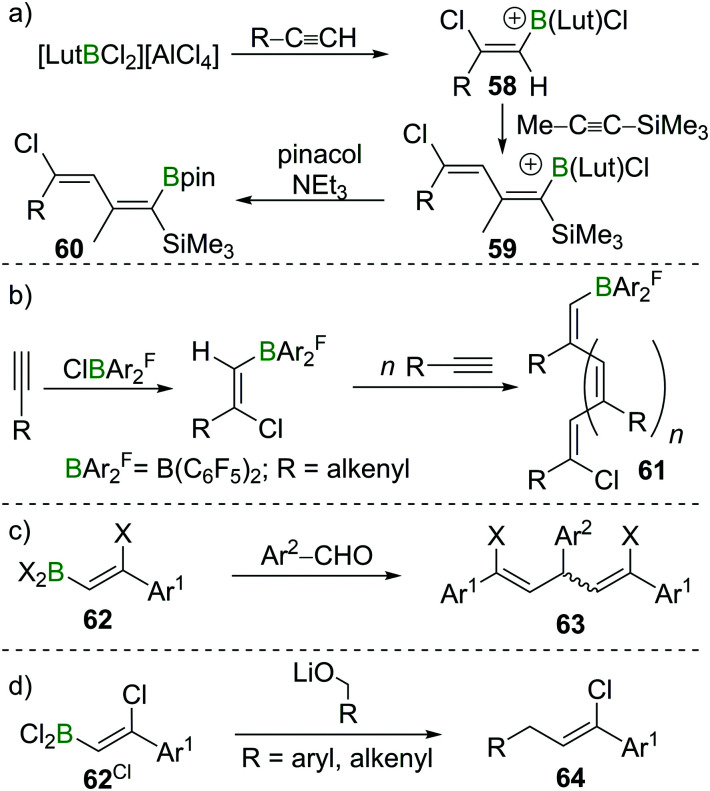
Select examples of functionalisation of haloborated alkynes *via* the B terminus.

The high B-centred Lewis acidity confers the alkenyl dihaloboranes a unique reactivity. The electrophilicity of the B atom paired with the nucleophilicity of the adjacent C atom enables the carboboration of alkynes. For example, the borocation [LutBCl_2_]^+^ (Lut = 2,6-lutidine) first reacts with R–C≡CH (R = alkyl, alkenyl, aryl) under 1,2-*syn*-haloboration to form compounds of general formula **58**. Upon addition of Me–C≡C–SiMe_3_, 1,2-*syn*-carboboration takes place, furnishing a borylated butadiene **59** which can be transformed into the corresponding pinacol ester by reaction with pinacol/NEt_3_ (**60**, Scheme 22a).^81^ Similar reactivity was observed for (F_5_C_6_)_2_BCl.^82^ If, however, (F_5_C_6_)_2_BX (X = Cl, Br) is reacted with enynes or cyclopropylacetylene, oligomerisation of the alkyne moiety to give **61** without interference of the alkene part of the molecule occurs. In this process, the newly formed C–B bond is added across the C≡C triple bond in a 1,2 carboboration reaction (Scheme 22b).^83^ Experiments by Eisch *et al.* suggest that haloboration is kinetically favoured over carboboration, but due to its irreversibility the latter is often the observed reaction outcome.^15^ Taking advantage of boron's intrinsically high oxophilicity, haloboration can be exploited to form 1,4-dienes from alkynes, BX_3_ and aldehydes under deoxygenative conditions in a one-pot process without isolation of the haloboration product **62** that is the intermediate in this process. Remarkably, the stereoselectivity is dependent on the halide employed with BCl_3_ furnishing exclusively the (*E*,*Z*) diastereomer and BBr_3_ the respective (*Z*,*Z*) isomer of **63** (Scheme 22c).^84,85^

In a similar fashion, propargyl,^86^ benzyl,^87^ or allyl^88^ alcohols can be used to access propargyl-, benzyl-, or allyl-substituted styrenes **64** (Scheme 22d). If instead of BX_3_ 9-I–BBN is used, haloboration of ethoxyethyne yields a haloboration product which reacts with aldehydes *via* a formal C=O carboboration. The resulting secondary alkylborinic esters are readily hydrolysed to α,β unsaturated carbonic esters.^89^

**Scheme 23:**
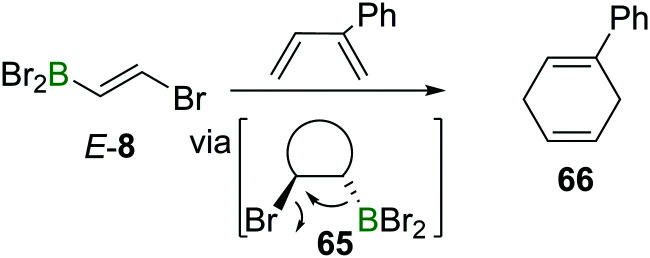
Diels–Alder reactions of haloboration products yield 1,4-cyclohexadienes.

The electron withdrawing nature of a BX_2_ group also renders alkenes like *E*-**8** electron poor, making them good substrates in Diels–Alder reactions. Due to the instability of α-halogenated alkylboranes (*cf.* Section 4), BBr_3_ elimination transforms the intermediate cyclohexene **65** into a 1,4-cyclohexadiene **66** (Scheme 23).^90^

### 6.1 Haloboration as a tool in natural product synthesis

**Scheme 24:**
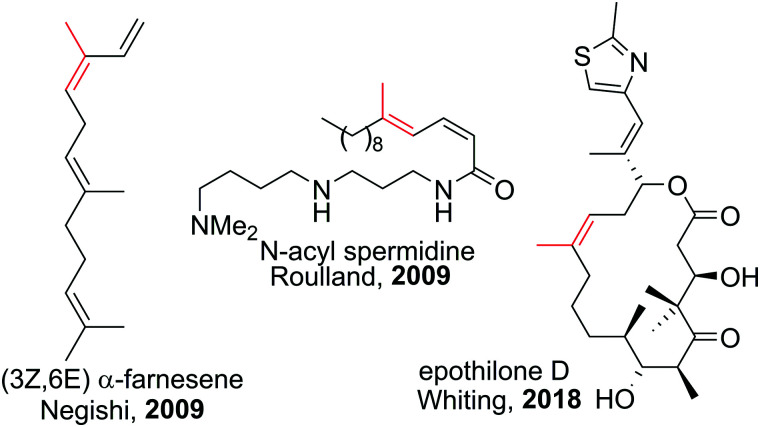
Select natural products synthesised *via* haloboration/cross-coupling sequences. The fragment incorporated in such manner is coloured in red.

Polyenes are common substructures in natural products (Scheme 24).^91,92^ Usually, the double bonds are formed *via* Wittig-type olefinations,^93^ and thus can suffer from forming mixtures of both *E* and *Z* isomers. Using the appropriate conditions the haloboration of terminal alkynes, as discussed above, proceeds stereoselectively (usually >98%^70,94,95^) and furnishes ambiphilic halogenated vinyl boronic acid derivatives post workup.

**Scheme 25:**
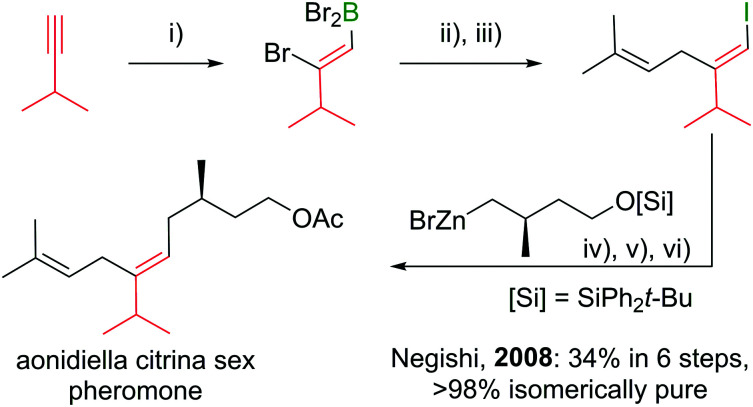
Example of a natural product synthesis sequence based on haloboration. *Reagents and conditions*: (i) BBr_3_, DCM, −78 °C to 23 °C, 1 h; (ii) Me_2_C=CH–CH_2_ZnBr, Pd(PPh_3_)_2_Cl_2_ (1 mol%), THF, 23 °C, 2 h; (iii) I_2_, NaOAc, THF/H_2_O, 23 °C, 1 h; (iv) zinc organyl, Pd(PPh_3_)_4_ (1 mol%), THF, 23 °C, 5 h; (v) [*n*-Bu_4_N]F, THF, 23 °C, 1 h; (vi) Ac_2_O, pyridine, DCM, 23 °C, 12 h.

Under the mild conditions of Pd catalysed cross-coupling reactions, the stereo information of the double bond is conserved.^94,95^ Thus, haloboration/cross-coupling sequences can be applied in the synthesis of olefinic natural products (Scheme 25).^96^ The inherent instability of polyene boronic acids can be circumvented by either using them directly without further purification or using the MIDA boronate derivatives. Indeed, only 12 different MIDA boronates derived *inter alia* from the haloboration product of acetylene are necessary to build most polyene natural products *via* consecutive Suzuki–Miyaura reactions.^75,92^

### 6.2 Haloboration in the synthesis of B-doped PAHs

**Scheme 26:**
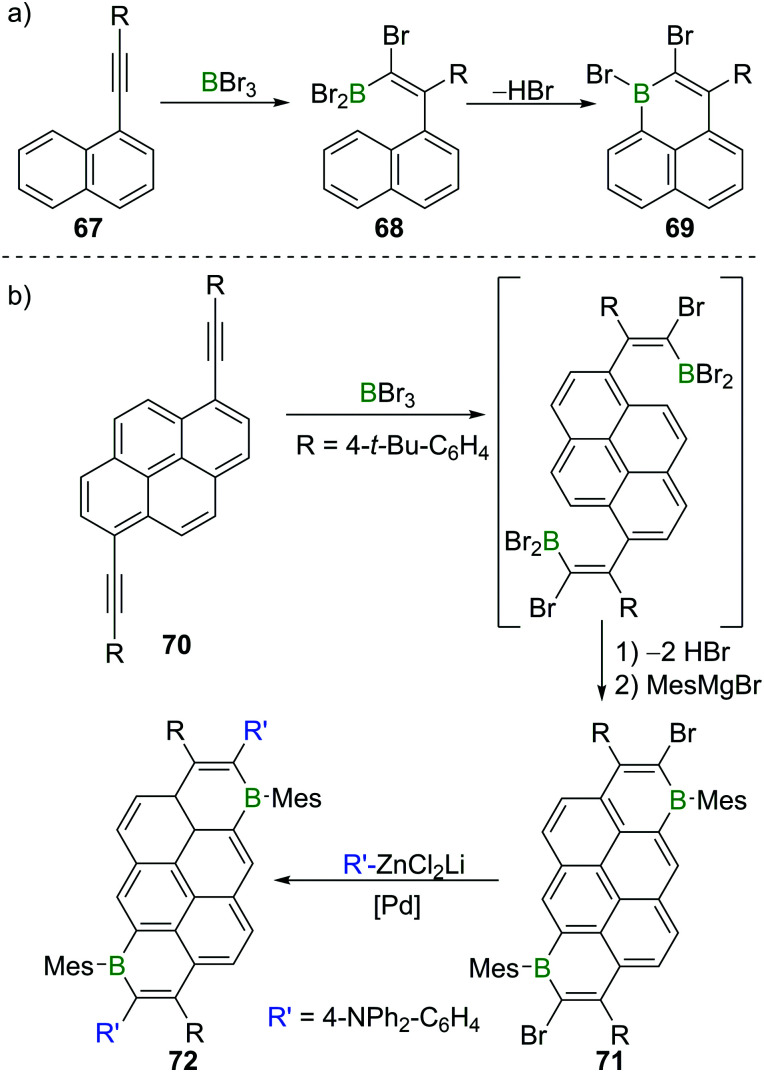
(a) Synthesis of 1-boraphenalene **69***via* haloboration/bora Friedel–Crafts reaction sequence. (b) Extension to pyrene derivatives.

Besides providing a useful route to form β-functionalised boronic-acid derivatives, haloboration is an expedient tool to form boron-doped polycyclic aromatic hydrocarbons (B-PAHs), which are promising candidate materials in the field of organic electronics as they are electron deficient aromatics with a comparatively low LUMO energy.^23,97^ Haloboration of 1-alkynylnaphthalenes **67** can lead selectively (under appropriate conditions) to either *trans*-1,2-haloboration products (R = H, alkyl) or 1,1-haloboration products **68** (R = aryl, Scheme 26), which then undergo bora Friedel–Crafts reactions furnishing 1-boraphenalenes (**69**, Scheme 26a).^97^ If pyrene instead of naphthalene is used as the hydrocarbon core (**70**), singly or doubly (**71**) B-doped PAHs with very low LUMO energies are accessible.^23^ The utility of bromoboration is then demonstrated by usage of the vinyl-Br unit in a subsequent Negishi coupling enabling donor–acceptor–donor complex **72** to be formed readily (Scheme 26b). In other materials synthesis applications the high Lewis acidity of BBr_3_ allows for a one-shot double cyclisation of *o*,*o*′-dimethoxy-substituted tolan derivatives by ether cleavage and concomitant *trans*-haloboration, yielding benzofurochromene derivatives (*cf.*Scheme 15, Section 3).^32^ In a similar fashion, *N*-protected propargylamines underwent intramolecular N–B bond formation post haloboration.^98^

**Scheme 27:**
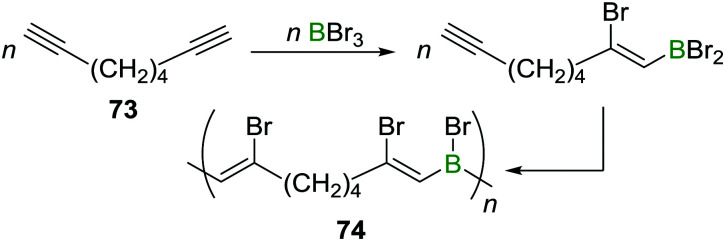
Haloboration of diynes can be used to construct B incorporating PAHs or to access poly(vinylboranes).

A related reaction sequence was used to construct even larger B,N doped PAHs.^99^ Besides being useful to incorporate boron into PAHs, haloboration has also been demonstrated to be a valuable tool to construct B-incorporated polymers. This was achieved by subjecting diynes such as **73** to multiple haloboration reactions using either BBr_3_ or R_2_BBr as the B source to yield polymers such as **74** (Scheme 27).^100^

## 7. Conclusion

Since its discovery almost 80 years ago, the haloboration of alkynes has developed into a reliable way to form bifunctional alkenes with excellent control of stereoselectivity possible. The haloboration of other substrates is more limited and further work is required to develop these into broadly useful transformations. Regarding alkyne haloboration, although some mechanistic details are still subject to debate, experimental evidence clearly demonstrates that the stereoselectivity of haloboration can be controlled. The addition of BX_3_ (X = Cl, Br, I) to a terminal alkyne proceeds *via syn*-addition of a B–X bond across the C≡C triple bond and is usually very fast, even at low temperatures. Thus, low-temperature quenching furnishes the *Z*-adduct selectively. At higher temperatures, isomerisation to the *E*-adduct can occur. The initial addition as well as the subsequent isomerisation are highly efficient, allowing access to the respective adduct in high yields with stereoselectivities often >98%. Haloboranes such as PhBBr_2_ or 9-Br–BBN can be employed in haloboration as well. For internal alkynes, stronger electrophiles than BCl_3_ are needed to effect haloboration, and most simply BBr_3_ can be used. The lower reactivity of internal C≡C triple bonds allows for the isolation of the *syn*-addition product even at room temperature within minutes. Yet, prolonged storage in solution or elevated temperatures (or more polar solvents) can lead to rearrangement of the 1,2-*syn* addition product to the 1,1-haloboration product in a reversible process. Thus, the addition of BBr_3_ to internal alkynes yields mixtures of both 1,1- and 1,2-adducts. Notably both the 1,2-adduct and the 1,1 adduct can be isolated selectively using the appropriate conditions.

The combination of halide and boron moieties in the addition product allows for broad diversification of the obtained olefin. For example, haloboration outperforms most other common C=C bond formation reactions in terms of selectivity and it has proved its worth in the field of polyene natural product synthesis. Notably, the primary products from haloboration contain vinylBX_2_ units which often have distinct reactivity compared to vinylB(OR)_2_ analogues due to the stronger electron withdrawing nature of the BX_2_ unit. An emerging application of the haloboration reaction is in the incorporation of B atoms into large delocalised π systems. This may either be achieved by using strategically positioned heteroatoms (*e.g.*, O, N) to direct alkyne haloboration, or by alkyne haloboration followed by a bora Friedel–Crafts reaction on alkyne-substituted PAHs. These are attractive as the formed B-doped PAHs are halogenated enabling facile subsequent diversification. We hope that further applications are forthcoming that help bring haloboration out of the shadow of the ubiquitous hydroboration reaction.

## Conflicts of interest

There are no conflicts to declare.

## Supplementary Material
